# Airborne LiDAR point cloud classification using PointNet++ network with full neighborhood features

**DOI:** 10.1371/journal.pone.0280346

**Published:** 2023-02-10

**Authors:** Xingzhong Nong, Wenfeng Bai, Guanlan Liu

**Affiliations:** 1 Guangzhou Metro Design & Research Institute Co., Ltd., Guangdong, Guangzhou, China; 2 School of Geodesy and Geomatics, Wuhan University, Wuhan, China; National University of Sciences and Technology, PAKISTAN

## Abstract

Compared with other point clouds, the airborne LiDAR point cloud has its own characteristics. The deep learning network PointNet++ ignores the inherent properties of airborne LiDAR point, and the classification precision is low. Therefore, we propose a framework based on the PointNet++ network. In this work, we proposed an interpolation method that uses adaptive elevation weight to make full use of the objects in the airborne LiDAR point, which exhibits discrepancies in elevation distributions. The class-balanced loss function is used for the uneven density distribution of point cloud data. Moreover, the relationship between a point and its neighbours is captured, densely connecting point pairs in multiscale regions and adding centroid features to learn contextual information. Experiments are conducted on the Vaihingen 3D semantic labelling benchmark dataset and GML(B) benchmark dataset. The experiments show that the proposed method, which has additional contextual information and makes full use of the airborne LiDAR point cloud properties to support classification, achieves high accuracy and can be widely used in airborne LiDAR point classification.

## Introduction

The airborne light detection and ranging (LiDAR) system provides a new technical approach for acquiring 3D spatial data, which provide reliable depth information. Airborne Laser Scanning (ALS) is one of the most import techniques for data collection for real-world scenes. The airborne laser scanning point cloud has been utilised in various fields, such as surveying and mapping, forestry survey [1], Underwater Navigation [2], 3D object detecting [3, 4], remote sensing [5], computer vision [6], disaster monitoring and cultural heritage protection [7].

The airborne LiDAR point has the following characteristics: (1) The categories in the airborne LiDAR point scenes have evident geometric properties. (2) The objects have extreme scale variations. (3) The objects have discrepancy distribution along the elevation [8]. Capturing high-resolution or fine-grained features for ALS point cloud classification is difficult because the geometric attributes are similar [9]. The early works for point cloud data processing focused on designing various handcrafted point descriptors derived from the surrounding neighbourhood [10, 11]. These point descriptors include density, roughness and curvature [12, 13]. The machine learning method is commonly used to achieve point cloud classification based on various handcrafted descriptors and various methods, e.g. support vector machine [14], random forest and AdaBoost [15]. Beside, Some probabilistic graphical models are used to take advantage of the contextual information [16, 17]. However, these methods heavily rely on handcrafted features and have limited generalisability for large-scale wild scenes. Nevertheless, the performance of the traditional method still heavily relies on the representation ability of the handcrafted features.

The deep learning method has recently achieved remarkable performance in scene classification, object detection and change detection [18]. Many researchers used deep learning–based methods to solve the problem for ALS point cloud classification. Some researchers projected row point clouds into 2D images [19] and then used convolutional neural networks (CNNs) for ALS classification to make full use of the advantages of CNNs [20]. The voxel-based method is another technique for ALS point cloud classification [21]. However, these projection methods usually require handcrafted features to enhance features and image representations, and the transformation from 3D to 2D inevitably cause information loss. In recent years, some studies have directly consumed raw point clouds and achieved state-of-the-art performance on some benchmarks.

The PointNet network [22] is a pioneering work that directly processes an irregular point. After the success of PointNet, many PointNet-like network architectures based on learned pointwise features have been proposed, such as PointNet++ [23], PointSift [24], PointCNN [25], PointWeb [26], DANCE-NET [27] and D-FCN [28]. Beside, the Graph-based method are develop rapidly [29, 30], such as LDGCNN [31] and Dynamic Graph CNN (DGCNN) [32]. ALS system has its own characteristics, including uneven point density, inexplicit structure and high redundancy. However, the works mentioned above have limitations and do not maximise the use of geometric structures. Thus, they lack the perception of geometry in encoded semantic features.

PointNet++ performs well on many tasks. However, its usage in large-scale airborne point clouds is not good. In PointNet++ network, the simple partitioning process cannot effectively capture a complicated relationship [33]. The recent advances in deep learning for point cloud processing are mainly focused on the designs of local aggregation operators. How to collect the information in the local area in the PointNet++ network still needs to be discussed.

In this study, we proposed a network modified from the PointNet++ network according to ALS point cloud characteristic. The main contributions of this study are shown as follows:

1. The proposed network can be trained in an end-to-end manner. The feature on the centroid point and the neighbour relationship in the sample layer on the PointNet++ network is added to improve the representation ability to solve the uneven distribution problem.
2. This study also utilises elevation information as weight on the up-sampling layer to support classification for the ALS point cloud, which exhibits discrepancies in elevation distributions.
3. The class-balanced term is used on the 3D point cloud to solve the highly uneven category distribution problem.

## Materials and methods

### Feature learning in local region

The PointNet++ network processes irregular point clouds directly, it is highly robust to small perturbations and occlusion. Thus, the need for expensive manual feature computation is eliminated, and a new solution for the 3D scene process is provided. The core of the PointNet++ network is the sampling layer, grouping layer and PointNet layer. The PointNet++ network uses FPS to choose point part on the sampling layer, the KNN method to partition the grouping layer and the PointNet to collect features on the PointNet layer.

Pointnet++ treats individual points in local point sets independently. This approach lacks the perception of the whole neighbourhood structure. The feature on the centroid in the PointNet++ network is assigned by the neighbourhood points. This data processing method possesses the following drawbacks: (1) Directly replacing the centre point feature with the neighbourhood feature makes the selection centroid point very critical. (2) Information interaction amongst neighbours’ points is lacking.

One point is not isolated to other points, and the attributes are formed by plenty of points [34]. Many features exist in the local region: neighbourhood features, centre point features and relationship features between neighbourhood points. In this chapter, the PointNet++ network is modified to enrich the local neighbourhood information description. Moreover, the centroid point information, neighbourhood point relationship and normalised coordinate information are combined. The formula for feature learning is shown as follows:

$$F_{i}=MLP(\Delta p_{ij}⊕{f^{\prime }}_{i}⊕F_{jj^{\prime }})|j,\;j^{\prime }\in N(i)$$ (1)

where Δ*p*_*ij*_ is the normalised coordinate information, $f_{i}^{\prime }$ is the centroid *i* information, and *F*_*jj*′_ is the relationship between neighbour point *j* and point *j*′. The *F*_*jj*′_ value can be obtained in many ways. The simplest way involves the maximum and minimum sum and average. However, this method destroys the module structure. Thus, we use the adaptive method to learn contextual information in local regions.

$$F_{jj^{\prime }}=\sum_{j^{\prime }=1}^{M}(W_{jj^{\prime }}•\Delta f_{jj^{\prime }})|j,j^{\prime }\in N(i)$$ (2)

where W_*jj*′_ is the adaptive weight, which is calculated from the difference between the two feature vectors, i.e. features *F*_*j*_ and *F*_*j*′_, after the MLP network. This adaptive weight uses convolution filters over neighbour point features and is shown as follows:

$$W_{jj^{\prime }}=MLP(F_{j}-F_{j^{\prime }})|j,j^{\prime }\in N(i))$$ (3)

The strategy to calculate adaptive weight W_*jj*′_ is also problematic because *W*_*jj*′_ is equal to zero when *j* = *j*′, indicating that the impact of *F*_*j*_ on itself is zero. To increase more learned information, we set the impact of *F*_*j*_ on itself is estimated by its own feature *F*_*j*_ when *j* = *j*′.

**Fig 1:**
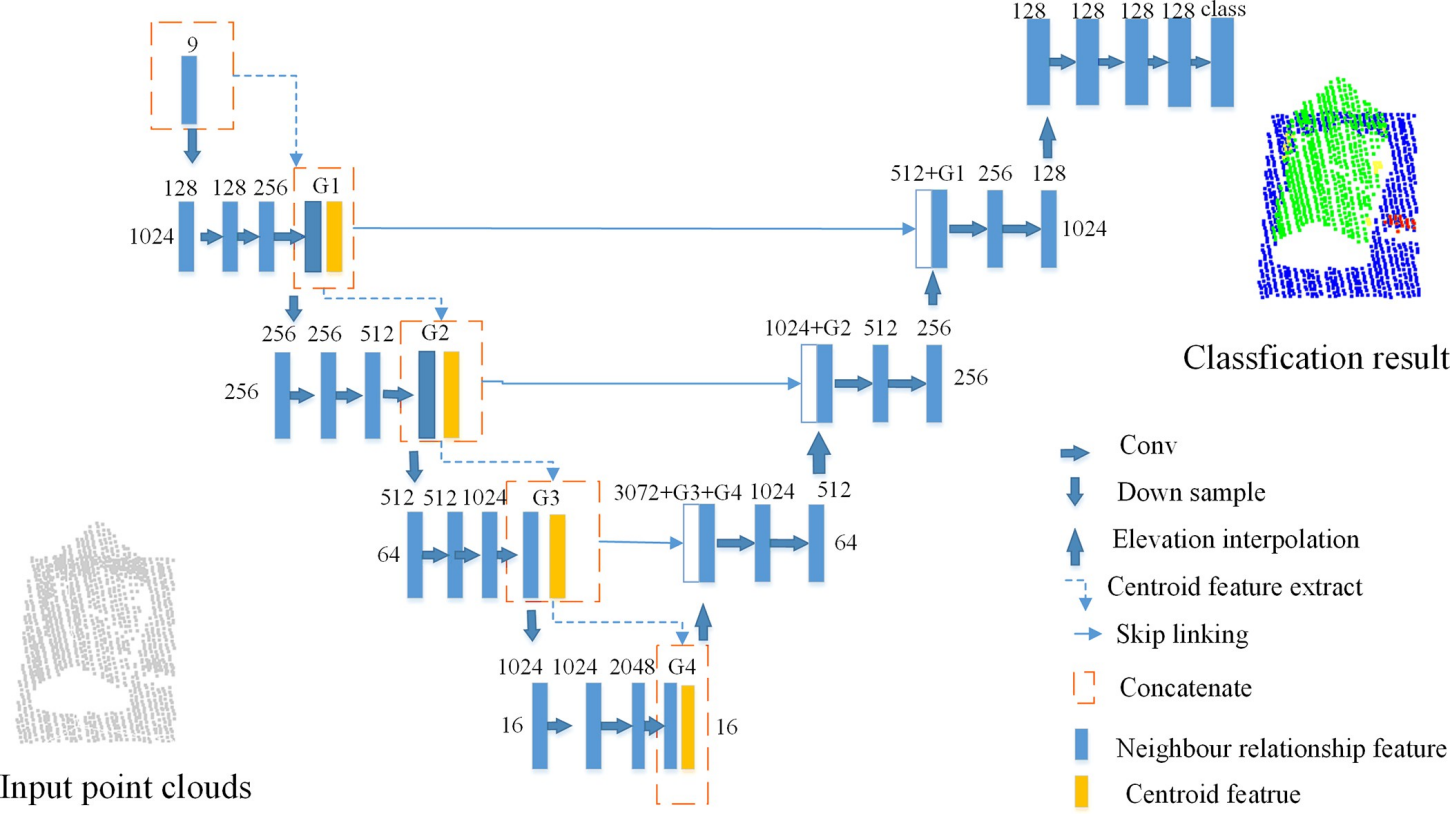
Overview of our proposed network architecture. This architecture added features on centroid and neighbour relationship. The input point clouds on the left are input points, and the output is the classification result.

The modified PointNet++ network has several abstraction layers and feature propagation layers with skip connections. The features on centroid and relationships amongst neighbours are also added. The architecture is shown in Fig 1.

### Class-balanced loss function

The category distribution in ALS scene is highly uneven which can be deduced from the point number in each category. The training efficiency is low when most locations do not provide useful learning signals. The challenge of long-tailed training data can be alleviated using two strategies: resampling and reweighting. In resampling, the number of examples for the minor class is oversampling, and the major class is under-sampling. However, the resampling always introduces a large amount of duplicated samples. Thus, the model becomes susceptible to overfitting, and the training slows down. Reweighting the loss by inverse class frequency usually yields poor performance for highly imbalanced classes. This strategy has poor performance on large-scale datasets.

Class-balanced terms perform well on 2D images. However, their performance on ALS point cloud classification is unknown. The class-balanced loss function formula is shown as follows:

$$CB(p,y)=-\frac{1-\beta }{1-\beta^{n_{y}}}L(p,y)$$ (4)

The main contribution of the class-balanced loss function is the added weighting factor $(1-\beta )/\left(1-\beta^{n_{y}}\right)$, where *n*_*y*_ is the number of samples in the ground truth class. When *β* = 0 corresponds to no reweighting, *β*→1 corresponds to reweighting by inverse class frequency. Therefore, adjusting the hyper-parameter *β* enables us to adjust the class-balanced term between no reweighting and reweighting via inverse class frequency.

The research shows that the class-balanced strategies perform remarkable improvements to existing commonly used loss functions, including sigmoid cross-entropy, softmax cross-entropy and focal loss [31]. The class-balanced softmax cross-entropy loss is

$$CB_{soft\max}(z,y)=-\frac{1-\beta }{1-\beta^{n_{y}}}\log(\frac{\exp(z_{y})}{\sum_{j=1}^{C}\exp(z_{j})})$$ (5)

The class-balanced sigmoid cross-entropy loss is

$$CB_{sigmoid}(z,y)=-\frac{1-\beta }{1-\beta^{n_{y}}}\sum_{i=1}^{C}\log(\frac{1}{1+\exp(-z_{i}^{t})})$$ (6)

The class-balanced focal loss is

$$CB_{focalloss}(z,y)=-\frac{1-\beta }{1-\beta^{n_{y}}}\sum_{i=1}^{C}{\left(1-p_{i}^{t}\right)}^{\gamma }\log(p_{i}^{t})$$ (7)

The class-balanced term is designed to address imbalanced data training, and can be applied to various deep networks.

### Adaptive elevation interpolation method

PointNet++ adopts a hierarchical propagation strategy with distance-based interpolation and across level skip links. The point feature propagation is achieved by interpolating feature values of *N*_*l*_ points at the coordinates of the *N*_*l*−1_ points. The weight on PointNet++ is the inverse distance based on *k* nearest neighbours. The ALS point clouds are discrepancy distribution along the elevation. For example, the point elevation on the ground is the lowest, followed by low plants, roofs and trees. The elevation information can effectively help distinguish different objects.

In this part, we embed the elevation information to the interpolation function to improve the performance on ALS point cloud classification further. The weight coefficient of distance is calculated based on the elevation difference between two neighbour points.

The point *i* interpolated features *f*^(*l*−1)^(*x*) are calculated from the neighbouring *j* point feature *f*^(*l*)^(*x*), which is shown as follows:

$$f^{\left(l-1\right)}(x)=\frac{\sum_{j=1}^{h}w_{ij}*f^{\left(l-1\right)}(x)}{\sum_{j=1}^{h}w_{ij}},j\in N_{i}$$ (8)

where *w*_*ij*_ denotes the weight coefficient of distance, which is shown as follows:

$$w_{ij}=\frac{w\_z_{ij}*d_{ij}{}^{2}}{\sum_{i=1}^{n}w\_z_{ij}}$$ (9)

where *d*_*ij*_ is the distance between points *i* and *j*, and *w*_*z*_*ij*_ is the weight coefficient of elevation, which is shown as follows:

$$w\_z_{ij}=\frac{1}{z_{ij}{}^{2}+{\bar{z}}^{2}}$$ (10)

where *z*_*ij*_ is the elevation diffident, and $\bar{z}$ is the average value of *z*_*ij*_. Then, the interpolated features are concatenated with skip linked point features from the subsampled features.

## Experimental result and analysis

**Fig 2:**
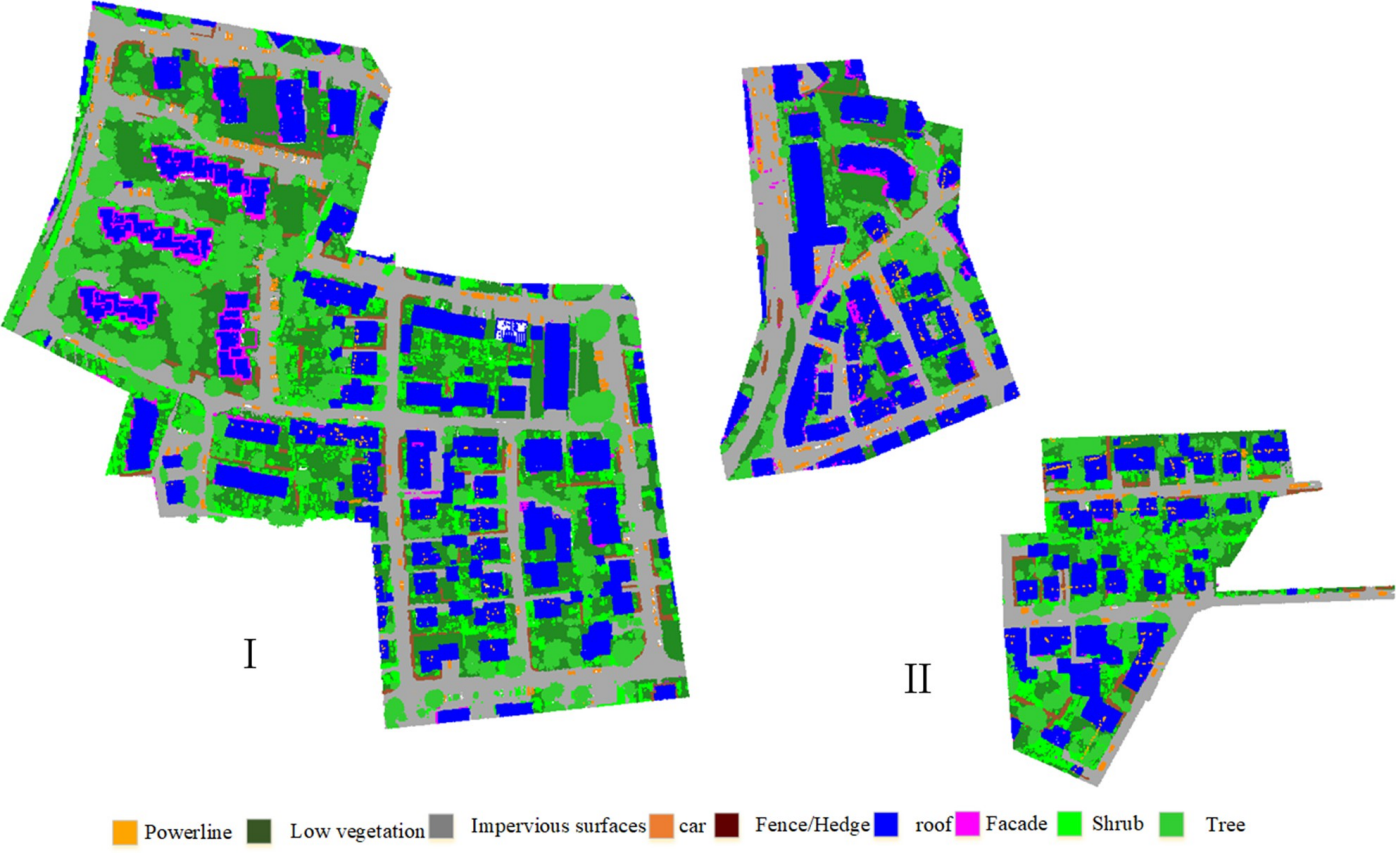
Overview of the ISPRS Vaihingen 3D labeling benchmark dataset. The legend at the bottom indicates the classification labels rendered in colours.

The experiments are conducted on two airborne LiDAR point datasets to evaluate the effectiveness of the proposed method. The first one is the Vaihingen 3D labeling benchmark dataset released by International Society for Photogrammetry and Remote Sensing (ISPRS). This dataset was acquired by an airborne Leica ALS50 system with a 45° field of view and an average flying height of 500 m. The point density on the no-overlapping area and cross-flight belt area is approximately 4 and 8 point/m^2^, respectively, indicating that the point in this dataset is uneven. The ALS scene is complex and possesses numerous geometric instances, complicating the automatic recognition of the model. The distribution of training sets and test sets is shown in Fig 2. All points in the Vaihingen dataset are annotated in nine semantic categories: power line, low vegetation, impervious surfaces, car, fence, roof, façade, shrub and tree. The overview of the ISPRS Vaihingen 3D dataset is shown in Fig 2.

The Vaihingen benchmark dataset contains three blocks. Scene (I) is used for training, and scene (II) is utilised for evaluation. The point data number for training is 753876, and the number for testing is 411722. The proportion in different categories is shown in Table 1.

**Table 1:** The percent of different categories in ISPRS vaihingen benchmark dataset.

categories	In training	In testing	categories	In training	In testing
Powerline	0.07	0.15	roof	20.17	26.48
Low_veg	23.99	23.97	Facade	3.61	2.72
Imp_surf	25.70	24.77	Shrub	6.31	6.03
car	0.61	0.90	Tree	17.9	13.17
Fence/Hedge	1.60	1.80			

Table 1 shows that the proportion of the power line category accounts for only 0.07%, whereas the impervious surfaces category accounts for 25.70%. The proportions in different categories are extremely uneven. Training directly on this dataset can be misleading.

Following the evaluation metric of the ISPRS 3D benchmark dataset, we use three metrics to evaluate our method, i.e. F1 score, Overall Accuracy (OA) and mean intersection over union (MIoU). MIoU is used to measure the classification performance for all categories. F1 score is calculated from the *precision* and *recall* values. It is suitable for classification evaluation in uneven class distribution. The calculations of precision, recall, F1 score and MIoU are formulated as (11)-(14):

$$precision=\frac{TP}{FP+TP}$$ (11)

$$recall=\frac{TP}{TP+FN}$$ (12)

$$F1=\frac{2*precision*recall}{precision+recall}$$ (13)

$$MIoU=\frac{1}{k+1}\sum_{i=0}^{k}\frac{TP}{FN+FP+TP}$$ (14)

where *TP*, *FN* and *FP* are the true positives, false negatives and false positives, respectively. The average precision *(AvgP)*, average recall *(AvgR)* and average F1 score *(AvgF1)* are also utilised.

### Model training

We train our model on a single NVIDIA Tesla V100 GPU. The proposed method is implemented using the PyTorch framework. Given the limited GPU memory, the training scene is divided into small patches with regular blocks with a size of 40 m * 40 m in the horizontal direction. The empirical knowledge of the model design is from reference [14] and comparative experiments. The parameters on sampling number, batch size, decay rate, training epoch, learning rate and optimiser are set as 4096, 3, 0.007, 64, 0.001 and Adam. The parameters in the training are saved every five epochs. For convenience, the validation dataset is set in the same manner as the training dataset. The MIoU is calculated regularly to validate the model. The highest MIoU is regarded as the best model. During the model testing, scene (II) is also segmented into 40 * 40 blocks in the horizontal direction, with a stride of 20 m. Then, the blocks are input into the best model to test the performance of our proposed method. We also test the loss function, adaptive elevation, adding feature and generalisation ability to investigate whether the ALS point cloud processing strategy is feasible.

### Test of the loss function

We first investigate the performance of our proposed method on the class-balanced term to select loss function. The ISPRS labelling dataset indicates that the point for each object category is quite different. Directly training on this unbalanced dataset may cause the classes with small numbers to become under fitted and misclassified. We use the modified loss function to address the issue mentioned by forcing our model to focus on the classes with few points. We experiment on the ISPRS dataset to investigate the performance of the class-balanced term and select the suitable function. The result is shown in Table 2.

**Table 2:** The comparison on different loss function module.

Loss function term	AvgP	AvgR	AvgF1	OA
Cross entropy	0.719	**0.696**	0.690	0.812
CB-sigmoid	0.741	0.672	0.694	0.817
CB-softmax,	0.742	0.649	0.681	0.819
CB-focalloss	**0.747**	0.681	**0.701**	**0.823**

From the value on Table 2 about AvgP, AvgR, AvgF1 and OA. the cross-entropy is low, and the focal loss function obtains a satisfying classification result. This result demonstrates that CB-focal loss obtains a quite stable performance. The CB-focal loss incorporates a category-specific weight factor for focal loss to reweight the classification loss. The class-balanced term combined with focal loss can outperform sigmoid cross-entropy and softmax cross-entropy. Therefore, we adopt CB-focal loss as the loss function for the following experiments.

### Test of adaptive elevation

The ALS point clouds are discrepancy distribution along the elevation. We investigate the effect of the adaptive elevation interpolation method by embedding the elevation information to the interpolation function to improve the performance on ALS point cloud classification. The weight coefficient of the distance is calculated based on the elevation difference between two neighbour points. The performance of this adaptive elevation is shown in Table 3.

**Table 3:** The comparison of adaptive elevation.

Interpolation method	AvgP	AvgR	AvgF1	OA
No adaptive elevation	0.747	0.681	0.701	0.823
Adaptive elevation	**0.757**	**0.687**	**0.705**	**0.831**

The AvgF1 and the OA improve by 0.4% and 0.8%, respectively. This finding indicates that the elevation information can effectively help distinguish different objects.

### Test of added information in the local region

We develop three models to investigate the effect of adding features about centroid and neighbour relationship on the subsample in the PointNet++ network. *Method (a)* is the model without the centroid and neighbour features. *Method (b)* is the model with centroid point features. *Method (c)* is the model with a neighbour relationship. The model with the features of centroid and neighbour relationship is marked as ‘*ours*’. The classification performance of these four models is listed in Table 4.

**Table 4:** The quantitative results using different features on local region.

Method	Powerline	Low_veg	Imp_surf	Car	Fence hedge	Roof	Façade	Shrub	tree	OA	AvgF1
(a)	0.718	0.815	0.910	0.765	0.388	0.922	0.570	**0.456**	**0.802**	0.831	0.705
(b)	**0.794**	0.817	0.911	0.788	0.370	**0.924**	0.602	0.427	0.788	0.829	0.713
(c)	0.683	**0.827**	**0.920**	**0.800**	**0.395**	0.919	**0.619**	0.419	0.783	0.833	0.707
Ours	0.776	**0.827**	0.917	0.792	0.389	0.922	0.613	0.432	0.791	**0.835**	**0.718**

Table 4 shows that the ALS point cloud classification performance is improved as the features become rich, and our proposed method has the highest OA and AvgF1. Method (a) and method (b) are limited for the feature extraction in distinguishing the classes in urban scenes because of scene complexity, unstructured nature of 3D point clouds, high sensor noise and incompleteness.

**Fig 3:**
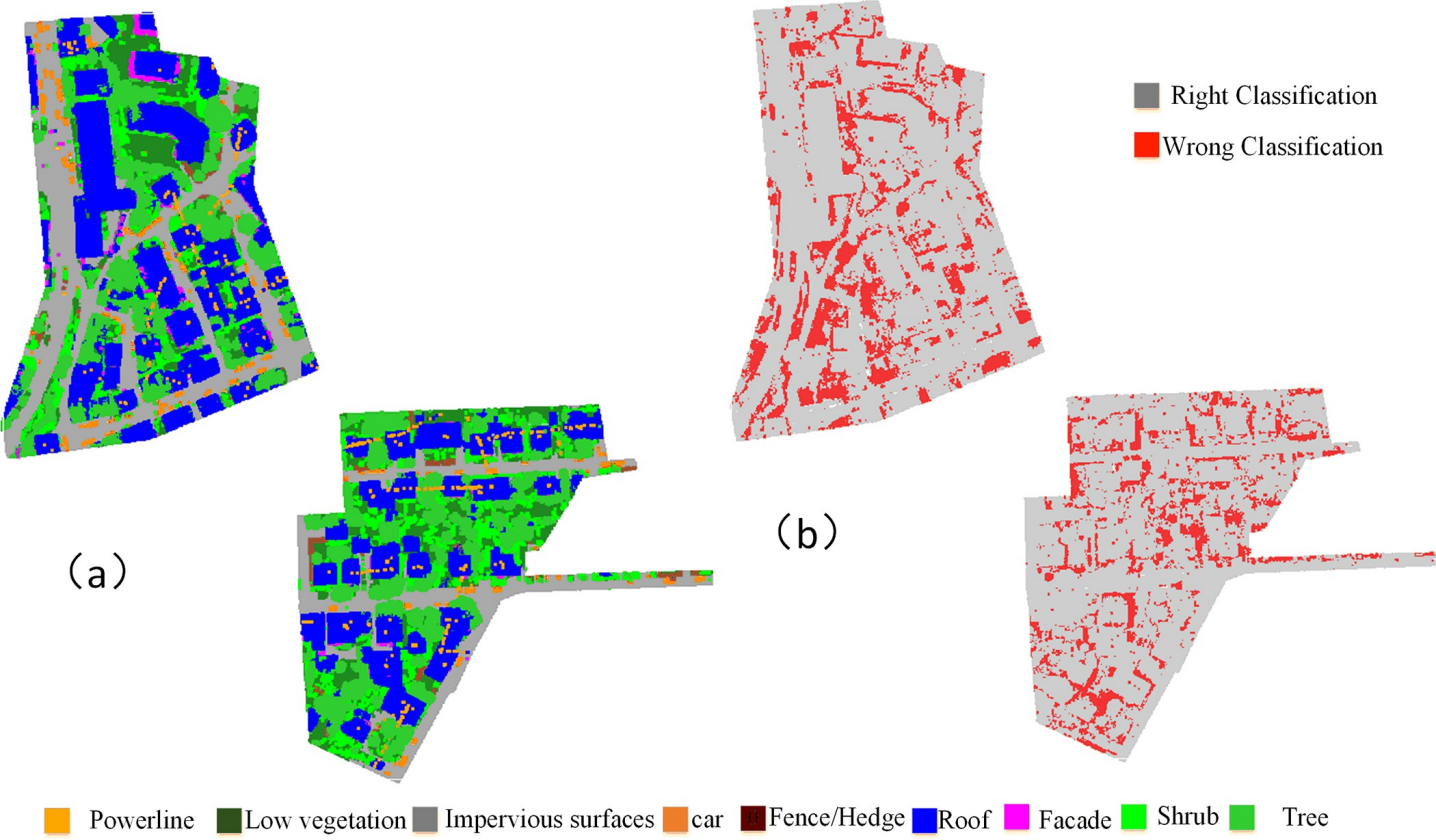
Prediction map (a) and error map (b) of our proposed method on the ISPRS benchmark dataset.

Our proposed method can effectively recognise most of the objects with an OA of 0.835 and AvgF1 0.718 by adding the feature about centroid point and neighbour relationship. The object class F1 scores that are higher than 60% are 7 out of 9, indicating that the proposed method can capture valuable features. Although the ISPRS dataset has a small number of points on the *power line* category, our proposed method achieves satisfying performance with an F1 score of 77.6%. Fig 3 shows the classification result of our proposed method and the error map.

Fig 3 shows that a large part of the test scenes is correctly classified. This observation is also validated by the error map. Table 5 shows the confusion matrix of the per-class accuracy of our proposed method.

**Table 5:** Classification confusion matrix of our proposed method. The evaluation metrics about precision, recall and F1 score of each class are reported. The numbers in the confusion matrix are normalised along each row.

Class	Powerline	Low_veg	Imp_surf	Car	Fence_hedge	Roof	Façade	Shrub	Tree
Powerline	0.778	0.000	0.000	0.000	0.000	0.171	0.027	0.008	0.017
Low_veg	0.000	0.842	0.052	0.003	0.009	0.017	0.010	0.055	0.012
Imp_surf	0.000	0.098	0.890	0.002	0.003	0.002	0.001	0.003	0.000
Car	0.000	0.023	0.006	0.903	0.004	0.001	0.007	0.038	0.018
Fence_hedge	0.000	0.074	0.003	0.013	0.747	0.002	0.003	0.107	0.051
Roof	0.001	0.011	0.002	0.001	0.002	0.951	0.014	0.010	0.007
Façade	0.001	0.014	0.003	0.001	0.004	0.186	0.714	0.037	0.038
Shrub	0.000	0.194	0.005	0.014	0.101	0.039	0.052	0.431	0.164
tree	0.001	0.024	0.002	0.001	0.022	0.108	0.021	0.102	0.719
precision	0.775	0.812	0.946	0.705	0.263	0.894	0.536	0.433	0.879
recall	0.778	0.842	0.890	0.903	0.747	0.951	0.714	0.431	0.719
F1 score	0.776	0.827	0.917	0.792	0.389	0.922	0.613	0.432	0.792

The confusion matrix shows that our proposed method obtains a quite satisfying performance on the impervious surfaces and power line categories as indicated by the F1 score. the proposed method also obtains acceptable performance on the roof and impervious surfaces categories. The shrub category and many shrub points are misclassified as low vegetation and tree points from the confusion matrix. The possible reason is that the shrubs have topological structures and elevation distribution similar to low vegetation and trees.

## Discussion

### Comparisons with other methods

We also compare our proposed method with other point-based models on the ISPRS 3D benchmark labelling. Table 6 lists the classification performance of our proposed method and all the methods compared.

**Table 6:** Performance comparison between our proposed method and other state-of-art supervised models on the ISPRS Vaihingen test dataset. The first nine columns in the table are the per-category F1 scores, and the last two columns are the OA and AvgF1.

Method	Powerline	Low_veg	Imp_surf	Car	Fence hedge	Roof	Façade	Shrub	tree	OA	AvgF1
PointNet	0.526	0.700	0.832	0.112	0.075	0.748	0.078	0.246	0.454	0.657	0.419
PointNet++	0.588	0.818	0.914	0.732	0.363	0.891	0.511	0.444	0.750	0.810	0.667
DGCNN	0.452	0.806	0.896	0.777	0.296	0.900	0.553	0.412	0.748	0.810	0.649
PointSift	0.577	0.807	0.909	0.778	0.305	0.925	0.569	0.444	0.796	0.822	0.677
D-FCN	0.704	0.802	0.914	0.781	0.370	0.930	0.605	0.460	0.794	0.822	0.707
PointCNN	0.615	**0.827**	**0.918**	0.758	0.359	0.927	0.578	0.491	0.781	0.833	0.695
PointCNN	0.630	0.826	0.919	0.749	**0.399**	**0.945**	0.593	**0.508**	0.827	**0.850**	0.711
+A-XCRF											
KPConv	0.631	0.823	0.914	0.725	0.252	0.944	0.603	0.449	0.812	0.837	0.684
our	**0.776**	**0.827**	0.917	**0.792**	0.389	0.922	0.613	0.432	0.792	0.835	**0.718**

Table 6 shows that the PointNet network has the lowest OA. The possible reason is that the PointNet network lacks neighbour information, and it does not collect enough features to represent the complex features on ALS point clouds. PointNet++ is a deep hierarchical network that recursively applies a unit PointNet on each grounded local region and makes full use of neighbour information. Compared with the baseline model (PointNet++ network), the proposed method increases by 2.5% in OA and 5.2% in AvgF1, which shows that the modified strategies are feasible.

DGCNN uses the dynamic graph convolutional neural to collect information. The collected neighbour point for centroid is the same. PointSift and D-FCN are the orientation-aware point feature learning methods. However, these methods ignore the relationship amongst neighbour points. PointCNN with A-XCRF [35] model obtains a high AvgF1. However, this model utilises a postprocessing step to refine the classification results. Our proposed method does not involve any postprocessing techniques.

Table 6 shows that the performance of our proposed method is better than the methods compared, as indicated by the AvgF1. Our proposed method also achieves remarkable higher performance on the powerline categories. The powerline category only takes a small proportion of the training data, which is difficult to predict, suggesting that the class-balanced term is feasible.

### Validation of generalisation ability

The GML(B) dataset are also conduct to validate the generalisation ability of our model, which belong to GML dataset for the B part and was acquired by the airborne Leica ALTM 2050 system. All points in this dataset are annotated in four semantic categories, including ground, building, tree and low vegetation. Each point contains only coordinate features. We use x, y, z coordinates as the model inputs in our experiments. Moreover, the hyperparameters are the same as the hyperparameters for the experiments on the Vaihingen dataset, except for the size of the segmented region, which is set as 48 m in the horizontal direction for the point density is approximately 7 point/m^2^, which is large than ISPRS benchmark dataset at 4–8 point/m^2^. We also compare the performance of our proposed method with that of PointNet, PointNet++ and the Random Forest on GML(B) benchmark labelling dataset. Table 7 lists the classification performance of our proposed method and all the methods compared.

**Table 7:** Quantitative comparisons between our proposed method and other models on the GML(B) benchmark dataset. The first four columns are the F1 scores for different classes, and the last two columns are the AvgF1 and compute time.

*method*	*Ground*	*Building*	*Tree*	*Low_veg*	*AvgF1*	*Time (h)*
PointNet	0.978	0.635	0.767	0.27	0.662	1.4
PointNet++	**0.980**	0.635	0.768	0.298	0.711	1.6
Random Forest	0.978	**0.816**	0.930	0.317	0.760	/
ours	0.979	0.746	**0.933**	**0.499**	**0.789**	**3.2**

Boldface text means the highest value in the column.

**Fig 4:**
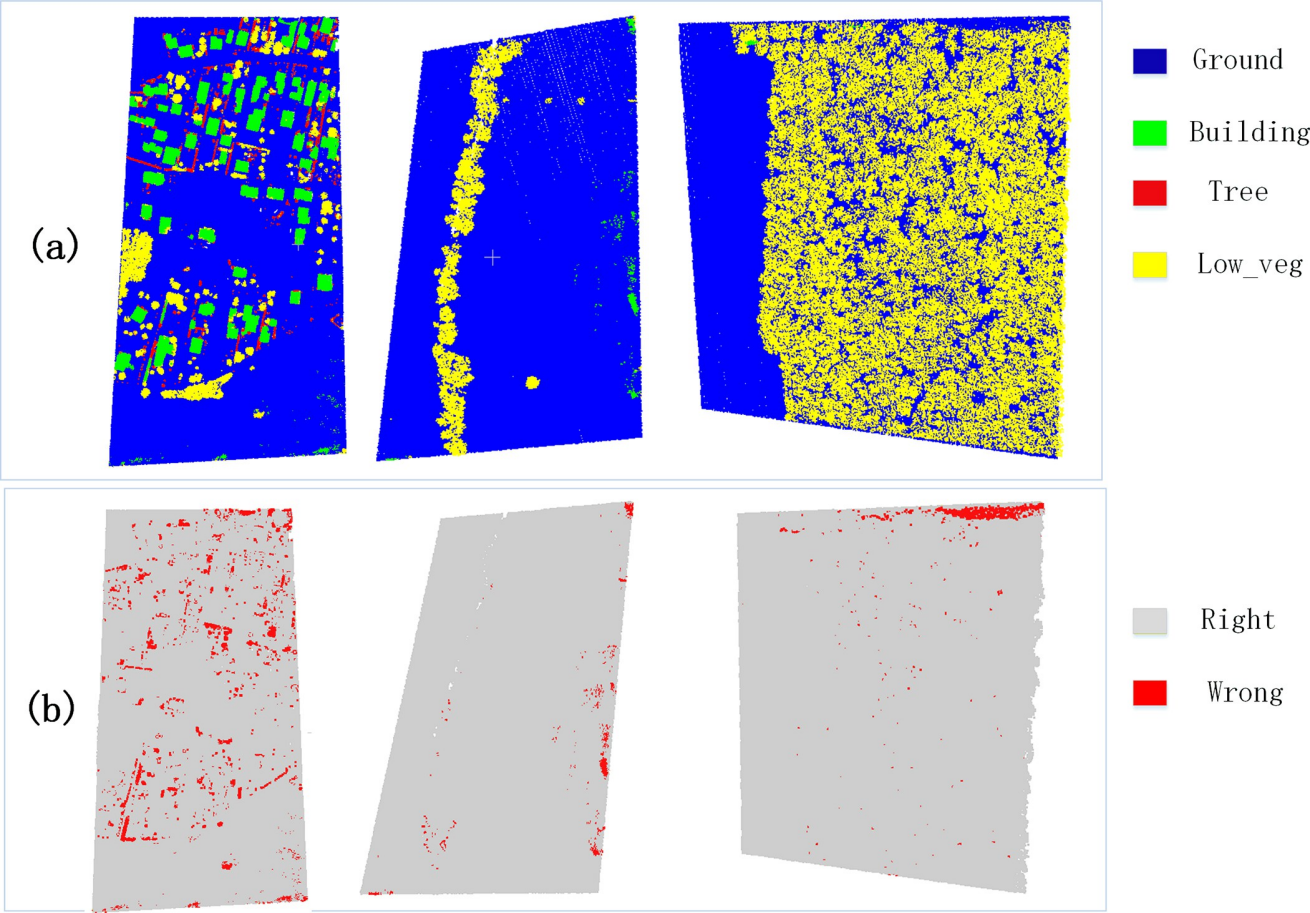
Prediction map (a) and error map (b) of our proposed method on the GML(B) benchmark dataset.

Table 7 shows that the ground, building and tree are well recognised. The worst classification result lies in the low vegetation. Our proposed method achieves the best performance in AvgF1. As for computing time comparison, PointNet network has the higher processing efficiency and the lowest precision, which indicates that lacking neighborhood features is not well to the recognition of objects. PointNet++ network still has low precision in large-scale complex scenes classification for this network still considers each point in the local region independently. Our proposed method has the lowest computational efficiency, which is mainly related to the time in extracting neighborhood point relationships and center point features. The result on Random Forest models is come from reference [36], which do not have compute time. However, the Random Forest model take each point’s local geometry independently and ignore the spatial dependencies. Fig 4 shows the classification result of our proposed method and the error map.

In Fig 4, many points are classified correctly, and errors are mainly distributed over the object edges. The modified PointNet++ network makes full use of receptive field information at all levels on the ALS point classification task. Our model can produce an accurate classification for the majority of ALS point clouds.

## Conclusion

This study proposes a modified PointNet++ network for airborne LiDAR point cloud classification based on their own characteristics, and verify the advantages of our proposed method through comprehensive ablation experiments on Vaihingen 3D semantic labelling benchmark dataset and the GML(B) dataset. Compared with the baseline model (PointNet++ network) from the Vaihingen datasets, the proposed method increases by 2.5% in OA and 5.2% in AvgF1, which shows that the modified strategies are feasible. Besides, the proposed method also achieves a new state-of-art performance for the power line category. The powerline category only takes a small proportion of the training data, which is difficult to predict, suggesting that the class-balanced term is feasible. The generalisation ability is verified by GML(B) dataset and our model can produce an accurate classification for the majority of ALS point clouds. The modified PointNet++ network makes full use of receptive field information at all levels and the elevation information can effectively help distinguish different objects on the ALS point classification task. Our method does not involve any postprocessing techniques and can operates unordered point sets with varying densities, which can be widely used in ALS point classification.
